# Allosteric activation of proto-oncogene kinase Src by GPCR–beta-arrestin complexes

**DOI:** 10.1074/jbc.RA120.015400

**Published:** 2021-01-13

**Authors:** Natalia Pakharukova, Ali Masoudi, Biswaranjan Pani, Dean P. Staus, Robert J. Lefkowitz

**Affiliations:** 1 Department of Medicine, Duke University Medical Center, Durham, North Carolina, USA; 2 Department of Biochemistry, Duke University Medical Center, Durham, North Carolina, USA; 3 Howard Hughes Medical Institute, Duke University Medical Center, Durham, North Carolina, USA

**Keywords:** Src, arrestin, G protein-coupled receptors, allosteric regulation, signal transduction, GPCR

## Abstract

G protein–coupled receptors (GPCRs) initiate signaling cascades via G-proteins and beta-arrestins (βarr). βarr-dependent actions begin with recruitment of βarr to the phosphorylated receptor tail and are followed by engagement with the receptor core. βarrs are known to act as adaptor proteins binding receptors and various effectors, but it is unclear whether in addition to the scaffolding role βarrs can allosterically activate their downstream targets. Here we demonstrate the direct allosteric activation of proto-oncogene kinase Src by GPCR–βarr complexes *in vitro* and establish the conformational basis of the activation. Whereas free βarr1 had no effect on Src activity, βarr1 in complex with M2 muscarinic or β2-adrenergic receptors reconstituted in lipid nanodiscs activate Src by reducing the lag phase in Src autophosphorylation. Interestingly, receptor–βarr1 complexes formed with a βarr1 mutant, in which the finger-loop, required to interact with the receptor core, has been deleted, fully retain the ability to activate Src. Similarly, βarr1 in complex with only a phosphorylated C-terminal tail of the vasopressin 2 receptor activates Src as efficiently as GPCR–βarr complexes. In contrast, βarr1 and chimeric M2 receptor with nonphosphorylated C-terminal tail failed to activate Src. Taken together, these data demonstrate that the phosphorylated GPCR tail interaction with βarr1 is necessary and sufficient to empower it to allosterically activate Src. Our findings may have implications for understanding more broadly the mechanisms of allosteric activation of downstream targets by βarrs.

G protein–coupled receptors (GPCRs), the largest group of membrane proteins, regulate virtually all physiological processes and represent the most common drug targets (1). GPCRs translate various extracellular stimuli into specific cellular responses via activation of signal transducers: heterotrimeric G-proteins and beta-arrestins (βarr). βarrs, initially discovered as proteins that desensitize G-protein signaling (2, 3), are now recognized as signal transducers in their own right (reviewed in Ref. 4).

βarr-dependent signaling begins with a two-step recruitment of βarr to the activated receptor. The first step involves binding to the phosphorylated receptor tail (5) (Fig. 1*A*) that converts βarr into an open active conformation characterized by, among other features, a 20-degree rotation between its N- and C-terminal domains (6). In addition, the C-edge loops of active βarr interact with the lipid bilayer and function as a membrane anchor (7, 8, 9). The second step involves the engagement of the finger-loop region of βarr with the receptor core (10) (Fig. 1*A*). The bimodal binding of βarrs to GPCRs engenders the coexistence of two unique conformations of GPCR–βarr complexes: the “tail” conformation and the “core” conformation, each with a distinct set of functions (11).

**Figure 1:**
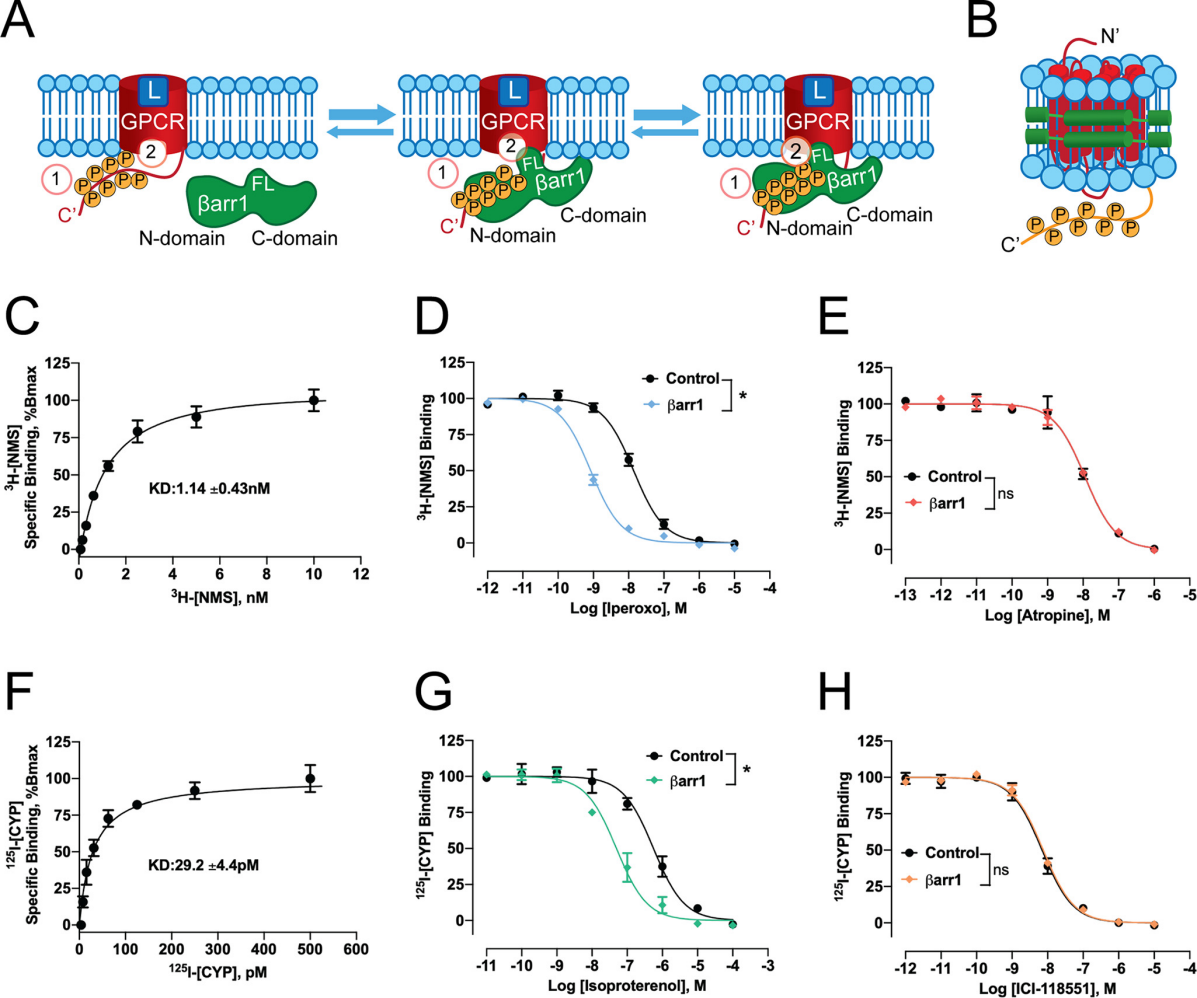
**Pharmacological characterization of M2V2 and β2V2 reconstituted in lipid nanodiscs: ligand binding and allosteric coupling of βarr1.***A,* schematic representation of GPCR–β-arrestin1 binding: βarr1 recruitment begins with binding to the phosphorylated receptor tail (*1*) and is followed by the engagement with the receptor core (*2*). C-edge loops of active βarr1 anchor it to the membrane (*L,* ligand; *FL,* finger loop). *B,* schematic representation of chimeric M2 muscarinic (M2V2) and β2-adrenergic (M2V2) receptors reconstituted in HDL particles (lipid nanodiscs). A synthetic phosphopeptide mimicking a phosphorylated C-terminal tail of V2 receptor was ligated to the receptors' C termini using sortase. The receptor is colored in *red*, the phosphorylated C-tail of V2 receptor is shown in *yellow*, MSP1D1E3 is shown in *green*. *C,* [^3^H]NMS saturation ligand binding at HDL-M2V2. *D* and *E,* competition ligand binding assays using [^3^H]NMS (1 nm) at HDL-M2V2 and a dose of agonist iperoxo (*D*) or antagonist atropine (*E*) in the absence (control) or presence of 1 μm βarr1. *F,*^125^I-CYP saturation ligand binding at HDL-β2V2. *G* and *H,* competition ligand binding assays using ^125^I-CYP (60 pm) at HDL-β2V2 and a dose of agonist isoproterenol (*G*) or antagonist ICI-118551 (*H*) in the absence (control) or presence of 1 μm βarr1. *C–H*, points in respective curves represent mean ± S.D. from three independent experiments. *Asterisks* (*) in *B* and *E* indicate significant difference in IC50 values between control *versus* βarr1 competition curves (*p* < 0.05, one-way ANOVA with Bonferroni's post test).

Upon activation by GPCRs, βarrs interact with a diverse set of partners including several mitogen-activated protein kinases and Src family tyrosine kinases (12, 13) among many others, and the physiological implications of these interactions are currently being explored in many laboratories. The nonreceptor tyrosine kinase Src is a pharmacologically important proto-oncogene, involved in the regulation of the cell cycle, adhesion, proliferation, and migration (reviewed in Ref. 14). It has been previously shown that βarr mediates the recruitment of Src to the activated β2-adrenergic receptor and thus functions as an adaptor protein (12). In fact, Src was the very first signaling protein for which βarr was shown to serve such an adaptor role. Moreover, recent data suggest the possibility of direct allosteric effects of βarr on Src (15, 16). However, the molecular mechanisms and the conformational basis of these effects have not been elucidated. Herein, we use purified proteins to evaluate whether βarr can directly allosterically activate Src and to explore the conformational basis of such regulation.

## Results

### GPCR–βarr1 complexes allosterically activate Src in vitro by promoting Src autophosphorylation

To study whether βarr1 directly activates Src *in vitro*, we expressed and purified Src, βarr1, and chimeric M2 muscarinic and β2-adrenergic receptors (M2V2 and β2V2, respectively). To ensure homogeneous phosphorylation, a synthetic phosphopeptide (V2Rpp) derived from the C-terminal tail of the vasopressin 2 (V2) receptor was ligated to the C termini of both receptors using sortase (17). V2Rpp has eight phosphorylated residues and confers tight binding to βarrs (6). M2V2 and β2V2 were reconstituted in high-density lipoprotein particles (HDL, lipid nanodiscs) as this environment closely mimics a native membrane and enables testing functional outcomes in response to different ligands (Fig. 1*B*). We chose ∼12–nm diameter MSP1D1E3 nanodiscs, previously found optimal for high-resolution structural studies of M2V2–βarr1 complex (9). The extended lipid surface of MSP1D1E3 enables anchoring of C-domain of βarr1 to the lipid bilayer, which stabilizes the complex (9).

Ligand binding and allosteric coupling of βarr1 to HDL-reconstituted receptors were verified by radioligand-binding assays (Fig. 1, *C*–*H*). [^3^H]NMS saturation ligand binding at HDL-M2V2 and ^125^I-CYP saturation ligand binding at HDL-β2V2 show apparent affinities of 1.14 ± 0.43 nm and 29.2 ± 4.4 pm for the respective radioligand (Fig. 1, *C* and *F*). Consistent with previous studies (17, 18), allosteric coupling of βarr1 results in a significant increase in agonist, but not antagonist, affinity at respective GPCRs (Fig. 1, *D, E,* G, and *H*).

**Figure 2:**
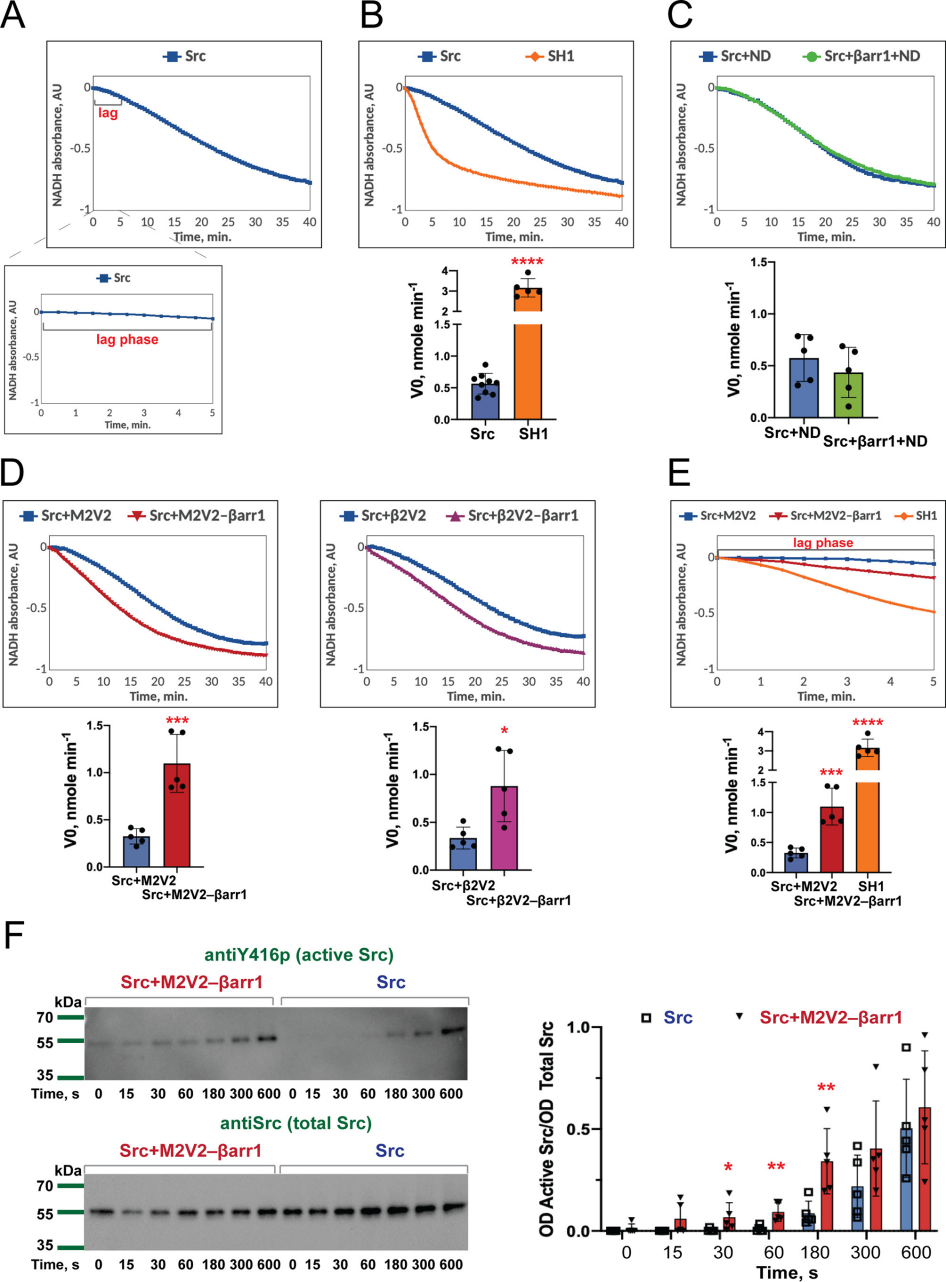
**GPCR-activated βarr 1 allosterically activates Src *in vitro* by reducing the lag phase in enzyme activation.***A,* lag phase in Src activation: representative progress curve of NADH oxidation coupled to peptide phosphorylation by Src measured by continuous kinase colorimetric assay shown over the course 40 min (*top panel*) and during the first 5 min of reaction (*bottom panel*). Optimal Src peptide (AEEEIYGEFEAKKKK) is used at a concentration of 250 μm, Src was used at a concentration of 25 nm. *B,* constitutively active Src (SH1) shows no lag phase. *Top panel*: representative progress curves of NADH oxidation coupled to peptide phosphorylation by Src and SH1 measured by continuous kinase colorimetric assay. *Bottom panel*: initial velocity of peptide phosphorylation (V_0_) by Src and SH1. Individual data show mean ± S.D. of five independent experiments (Src: 9 independent experiments). Statistical differences were determined by one-way ANOVA and Dunnett's multiple comparison test (****, *p* < 0.0001). Optimal Src peptide (AEEEIYGEFEAKKKK) is used at a concentration of 250 μm, Src and SH1 were used at a concentration of 25 nM. *C,* βarr1 alone does not have an effect on Src activity. *Top panel*: representative progress curves of NADH oxidation coupled to peptide phosphorylation by Src as measured by continuous kinase colorimetric assay. *Bottom panel*: initial velocity of peptide phosphorylation (V_0_). Individual data show mean ± S.D. of five independent experiments. Statistical differences were determined by one-way ANOVA and Dunnett's multiple comparison test. Optimal Src peptide (AEEEIYGEFEAKKKK) is used at a concentration of 250 μm, Src was used at a concentration of 25 nm, βarr1 was used at a concentration of 125 nm. To reproduce the exact conditions of the experiment with GPCR–βarr1 complexes, empty MSP1D1E3 (*ND*) nanodisc and Fab30 were added to Src at a concentration of 125 nm. *D,* M2V2–βarr1 and β2V2–βarr1 complexes activate Src *in vitro. Top panel*: representative progress curves of NADH oxidation coupled to peptide phosphorylation by Src as measured by continuous kinase colorimetric assay. *Bottom panel*: initial velocity of peptide phosphorylation (V_0_). Individual data show mean ± S.D. of five independent experiments. Statistical differences were determined by one-way ANOVA and Dunnett's multiple comparison test (*, *p* < 0.05; ***, *p* < 0.0005). Optimal Src peptide (AEEEIYGEFEAKKKK) was used at a concentration of 250 μm and Src was used at a concentration of 25 nm. M2V2–βarr1 and β2V2–βarr1 are used at a concentration of 125 nm. M2V2–βarr1 and β2V2–βarr1 complexes are additionally stabilized by a synthetic antibody fragment Fab30 (125 nm). To reproduce the exact conditions of the experiment with GPCR–βarr1 complexes, M2V2/β2V2 and Fab30 were added to Src at a concentration of 125 nm. M2V2 was activated by iperoxo, β2V2 was activated by BI-167107. *E,* GPCR-activated βarr1 reduces the lag phase in enzyme activity: *Top panel*: representative progress curves of NADH oxidation coupled to peptide phosphorylation by Src as measured by continuous kinase colorimetric assay during first 5 min of reaction. *Bottom panel*: initial velocity of peptide phosphorylation (V_0_). Optimal Src peptide (AEEEIYGEFEAKKKK) is used at a concentration of 250 μm, Src and SH1 were used at a concentration of 25 nm. M2V2–βarr1 is used at a concentration of 125 nm. M2V2-βarr1 complex is additionally stabilized by a synthetic antibody fragment Fab30 (125 nm). M2V2 was activated by iperoxo. To reproduce the exact conditions of the experiment with GPCR–βarr1 complexes, M2V2/β2V2 and Fab30 were added to Src at a concentration of 125 nm. M2V2 was activated by iperoxo, β2V2 was activated by BI-167107. *F,* M2V2–βarr1 complex promotes Src autophosphorylation. *Left panel*: time course of activation loop Tyr-416 autophosphorylation of Src *in vitro*. Representative Western blots are shown. *Right panel*: densitometry analysis of Tyr-416 phosphorylation expressed as a ratio over total Src (optical density of active Src/OD total Src). Src was used at a concentration of 12.5 nm and M2V2–βarr1–Fab complexes were used at a concentration of 125 nM. Individual data show mean ± S.D. of five independent experiments. Statistical differences were determined by Mann-Whitney test (*, *p* < 0.05; **, *p* < 0.01 as compared with the corresponding time point of Src alone reaction).

We then measured the rate of phosphorylation of a synthetic peptide substrate (AEEEIYGEFEAKKKK) by Src using a continuous kinase colorimetric assay (19). In this assay the rate of peptide phosphorylation is coupled via the pyruvate kinase/lactate dehydrogenase enzymes to the oxidation of NADH measured through the decrease in absorbance at 340 nm. A progress curve of Src begins with a short lag phase with no or little changes in NADH absorbance (Fig. 2*A*). This lag phase is associated with the slow activation step caused by the disruption of an autoinhibited conformation of Src and intermolecular autophosphorylation of catalytic Tyr-416 (19). Once Src is fully activated, it quickly phosphorylates the substrate, which causes a rapid decrease in absorbance (Fig. 2*A*). The presence of lag phase in Src activity is clearly illustrated by comparing the progress curves of WT Src and a purified kinase domain of Src (SH1). SH1 represents a constitutively active form of the enzyme (20) and demonstrates no lag phase (Fig. 2*B*). Due to the presence of the slow activation step, the Src kinetics before and during the activation process is divergent from Michaelis-Menten kinetics. Because the lag phase is indicative of the degree of autoinhibition of the enzyme, the initial velocity of the reaction (V0) represents the most accurate parameter to measure Src activity (21, 22).

We then performed the assay in the presence of βarr1 and GPCR–βarr1 complexes. Whereas free βarr1 did not affect Src activity (Fig. 2*C*), βarr1 in complex with either phosphorylated M2V2 or β2V2 activated by iperoxo or BI-167107, respectively, caused a significant increase in the rate of peptide phosphorylation by Src (Fig. 2*D*, Table S1). Addition of GPCR–βarr1 complexes reduces this lag phase and leads to a more rapid decrease in absorbance (Fig. 2*E*). M2V2 and β2V2 alone did not lead to Src activation (Fig. 2*D*) indicating that this process is mediated by βarr1. The initial velocity of peptide phosphorylation by SH1 is 5.6-fold higher than that of WT Src (3.16 ± 0.45 *versus* 0.56 ± 0.16 nmol min^−1^, respectively), whereas in the presence of M2V2–βarr1 and β2V2–βarr1 we observed a 3.4- and a 2.6-fold increase in the initial rate, respectively (Table S1). Taken together, our data demonstrate that receptor stimulated βarr1 directly activates Src.

Kinase assay data suggest that GPCR–βarr1 complexes reduce the lag phase in Src activation (Fig. 2*E*). We thus hypothesized that interactions of GPCR–βarr1 complexes with Src promotes Src autophosphorylation. To test this hypothesis, we analyzed the time course of Tyr-416 autophosphorylation by Western blotting (Fig. 2*F*). In the presence of the agonist-activated M2V2–βarr1 complex, a phosphorylation of Tyr-416 was observed within 15-30 s after adding ATP, whereas for the Src alone reaction autophosphorylation was detected only at later time points. These data suggest that GPCR-stimulated βarr1 activates Src by reducing the lag phase in Src autophosphorylation.

### Phosphorylated GPCR tail interaction with β-arrestin 1 is sufficient to confer the activation of Src

**Figure 3:**
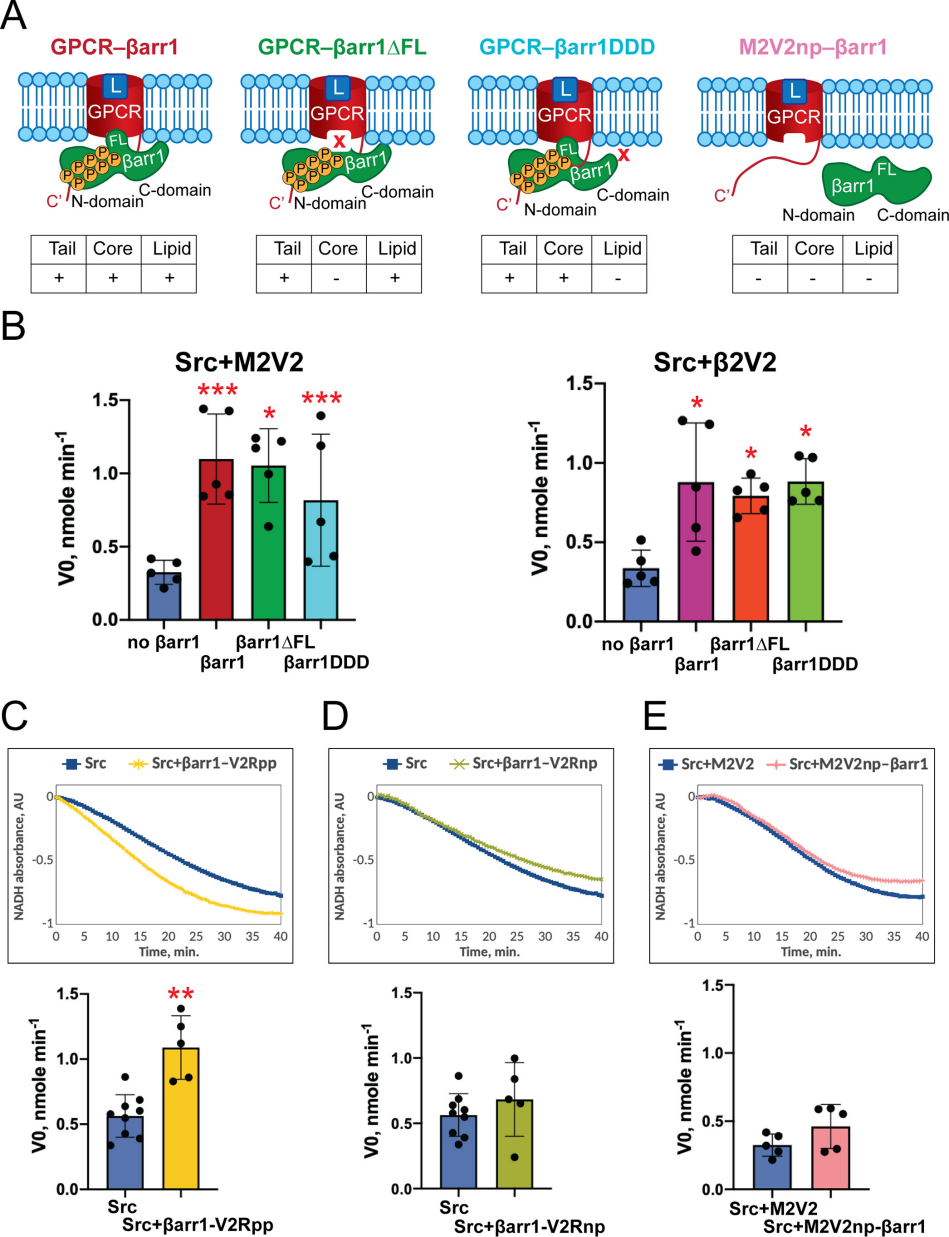
**Phosphorylated GPCR tail interaction with** β**-arrestin 1 is sufficient to confer the activation of Src.***A,* cartoon illustrating the effect of βarr1 mutations and receptor C-terminal tail phosphorylation on the conformation of GPCR–βarr1 complexes. GPCR–βarr1 complexes are additionally stabilized by a synthetic antibody fragment Fab30 (not shown for clarity) (*L,* ligand; *FL,* finger loop). *B,* interactions of βarr1 with the receptor core and with the lipid are dispensable for activation of Src. Initial velocity of peptide phosphorylation by Src as measured by continuous kinase colorimetric assay. Individual data show mean ± S.D. of five independent experiments. Statistical differences were determined by one-way ANOVA and Dunnett's multiple comparison test (*, *p* < 0.05; ***, *p* < 0.0005). Optimal Src peptide (AEEEIYGEFEAKKKK) was used at a concentration of 250 μm and Src used at a concentration of 25 nm. M2V2–βarr1 and β2V2–βarr1 are used at a concentration of 125 nm. M2V2–βarr1 and β2V2–βarr1 complexes are additionally stabilized by a synthetic antibody fragment Fab30 (125 nm). To reproduce the exact conditions of the experiment with GPCR–βarr1 complexes, M2V2/β2V2 and Fab30 were added to Src at a concentration of 125 nm. M2V2 was activated by iperoxo, β2V2 was activated by BI-167107. *C–E,* receptor C-terminal tail phosphorylation is required for allosteric activation of Src. Representative progress curves of NADH oxidation coupled to peptide phosphorylation by Src, as measured by continuous kinase colorimetric assay (*top panel*) and initial velocity of peptide phosphorylation by Src (*bottom panel*), in the presence of βarr1–V2Rpp (*C*), βarr1–V2Rnp (*D*), or M2V2np–βarr1 (*E*) are shown. Individual data show mean ± S.D. of five independent experiments (Src: 9 independent experiments). Statistical differences were determined by one-way ANOVA and Dunnett's multiple comparison test (**, *p* < 0.01). Optimal Src peptide (AEEEIYGEFEAKKKK) is used at a concentration of 250 μm and Src was used at a concentration of 25 nm. βarr1–V2Rpp, M2V2np, βarr1, V2Rnp, and Fab30 are added at a concentration of 125 nm. To reproduce the exact conditions of the experiment with GPCR–βarr1 complexes, M2V2 and Fab30 were added to Src at a concentration of 125 nm. M2V2np was activated by iperoxo.

We next sought to elucidate which conformations of GPCR–βarr complexes contribute to Src activation. Previously shown that β2V2–βarr1 complex represents a dynamic mixture of tail (partially engaged) and core (fully engaged) conformations (10). Recent structural and biophysical data demonstrate that in addition to tail and core interactions with the receptor, the C-edge of active βarr also engages the lipid nanodisc and functions as a membrane anchor (Figs. 1*A* and 3*A*) (8, 9).

First, we tested whether an interaction of βarr1 with the receptor core is required for Src activation. Importantly, because C termini of both M2V2 and β2V2 are phosphorylated, βarr1 will bind to the receptor tail regardless of the presence of an agonist. Interestingly, we observed full activation of Src even without agonist stimulation of β2V2 (Fig. S1). This result suggests that allosteric activation of Src is primarily driven by interaction of βarr1 with the phosphorylated receptor tail. However, existing evidence of high (basal) constitutive activity of GPCR reconstituted in lipid nanodiscs (23) required additional experiments to verify this hypothesis.

To prevent βarr coupling to the receptor core, we expressed and purified a βarr1 mutant with a deleted finger loop (βarr1ΔFL). βarr1ΔFL is unable to interact with the receptor core (11), thus all GPCR–βarr1ΔFL complexes will remain in the tail conformation (Fig. 3*A*). Interestingly, both M2V2–βarr1ΔFL and β2V2–βarr1ΔFL complexes fully retain the ability to activate Src suggesting that core interaction is dispensable for allosteric activation of the kinase (Figs. 3*B*).

We next tested whether the engagement of βarr1 with the lipid bilayer plays a role in βarr1 ability to activate Src. Molecular dynamic simulations have shown that interactions between C-edge loops of βarr1 and the membrane stabilize the active conformation of βarr1 (9) that might be crucial for allosteric activation of Src. We thus formed GPCR–βarr complexes with a βarr1 mutant deficient in lipid interaction (βarr1DDD) (9) (Fig. 3*A*) and measured Src activity. M2V2–βarr1DDD and β2V2–βarr1DDD complexes activate Src as efficiently as complexes formed with WT βarr1 and βarr1ΔFL indicating that anchoring of βarr1 to the membrane is inessential for allosteric activation of the enzyme (Figs. 3*B*).

Our findings thus suggest that the interaction of βarr1 with a phosphorylated receptor tail is sufficient to allosterically activate Src. We then tested if active βarr1 with only a phosphorylated C-terminal tail of the V2 receptor (βarr1–V2Rpp) induces activation of Src similarly to GPCR–βarr1 complexes. Indeed, we achieved the same level of Src activation in the presence of βarr1–V2Rpp and GPCR–βarr1 (Fig. 3*C*, Table S1).

We next sought to ascertain whether phosphorylated GPCR tail interaction with βarr 1 is absolutely required for allosteric activation of Src. We thus tested Src activity in the presence of βarr1 and nonphosphorylated C-terminal tail of the V2 receptor (V2Rnp) (Fig. 3*D*). As expected, no significant increase in Src activity was observed (Fig. 3*D*). Furthermore, the presence of βarr1 and nonphosphorylated M2V2 receptor (M2V2np) (Fig. 3*A*) also did not activate Src (Fig. 3*E*), probably due to the reduced binding of βarr1 to the nonphosphorylated receptor. To test the binding of βarr1 to both M2V2 and M2V2np, we performed a M1-FLAG pulldown assay (Fig. S2). Even though M2V2np binds a small amount of βarr1, it is not sufficient to trigger the activation of Src, as the large portion of βarr1 predominantly remains in an inactive conformation. Taken together, these data indicate that the phosphorylated GPCR tail interaction with βarr1 is necessary and sufficient to drive the allosteric activation of Src.

### β*-Arrestin 1 mediates allosteric activation of Src by interacting with SH3 domain*

**Figure 4:**
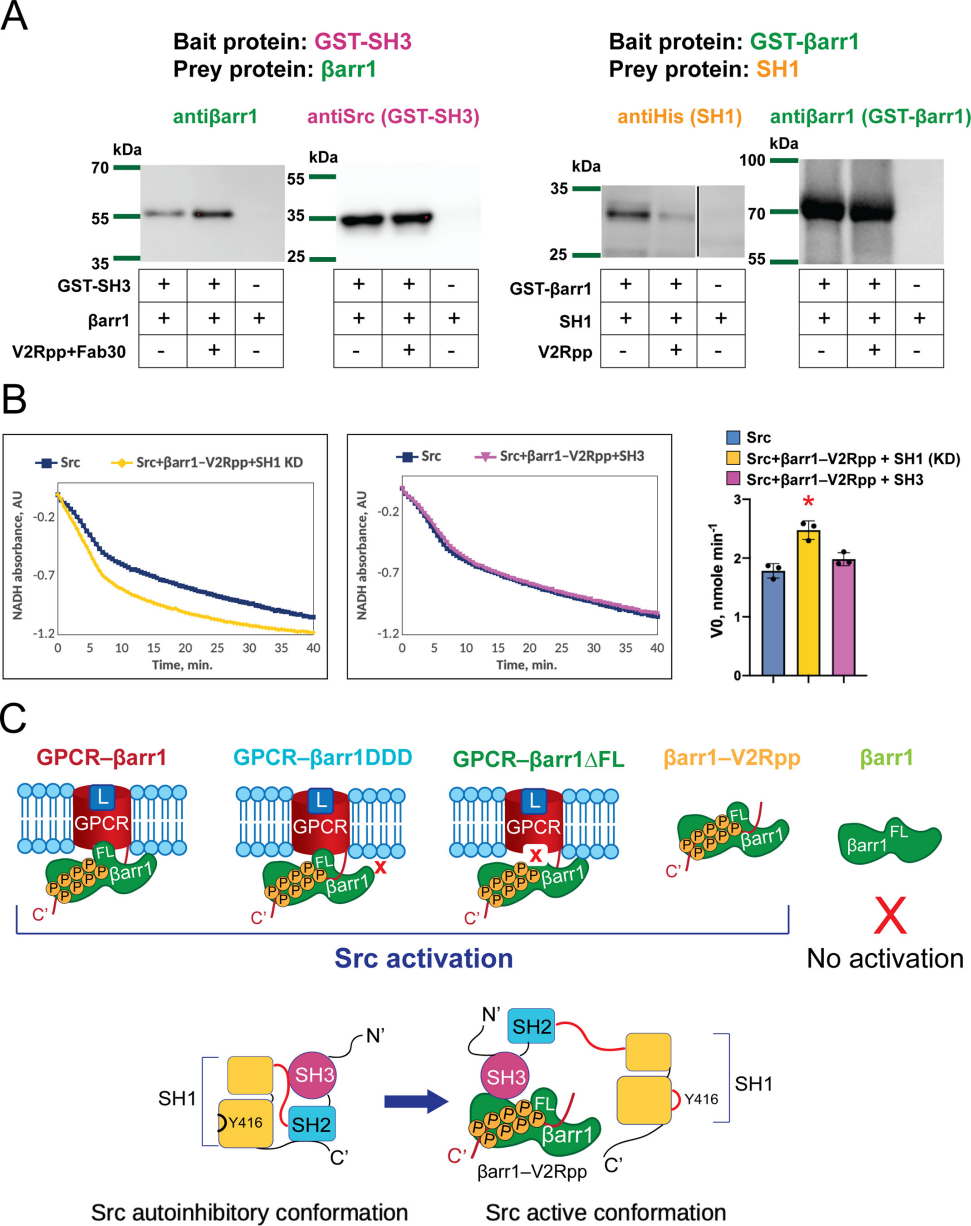
**GPCR-activated βarr1 activates Src by interacting with its SH3 domain.***A,* βarr1 interacts with SH3 and SH1 domains of Src. *Left panel*, GST-pulldown assay of SH3 domain and βarr1. *Right panel*, GST-pulldown assay of SH1 domain and βarr1. The *right lane* separated by a *black line* was spliced from a non-neighboring lane of the same blot. Data shown are representative of three independent experiments. *B,* addition of an excess of SH3 domain blocks βarr1-mediated activation of Src. *Left* and *middle panels show* representative progress curves of NADH oxidation coupled to peptide phosphorylation by Src in the presence of βarr1–V2Rpp and excess SH1 kinase dead (*SH1 KD*) (*left panel*) or SH3 domains (*middle panel*) measured by continuous kinase colorimetric assay. *Right panel*, initial velocity of peptide phosphorylation by Src in the presence of βarr1–V2Rpp and excess of SH3 or SH1 KD domains. Individual data show mean ± S.D. of three independent experiments. Statistical differences were determined by Kruskal-Wallis test (one-way ANOVA on ranks) and Dunn's multiple comparison test (*, *p* < 0.05 as compared with Src alone). Optimal Src peptide (AEEEIYGEFEAKKKK) is used at a concentration of 250 μm, Src is used at a concentration of 200 nm, SH3 and SH1 KD are used at a concentration of 6 μm. βarr1–V2Rpp (stabilized by Fab30) is used at a concentration of 1.2 μm. *C,* conformational basis of βarr1-mediated activation of Src. Various tail-bound βarr1 conformations interact with SH3 domain of Src and disrupt the autoinhibited conformation of the enzyme (*L,* ligand; *FL*, finger loop).

Our findings demonstrate that active βarr1 mediates allosteric activation of Src by promoting autophosphorylation of the enzyme. We next wanted to delineate the molecular mechanism of the activation. First, we tested the binding of active and inactive conformations of βarr1 to different regions of Src *in vitro* using a GSH *S*-transferase (GST)-pulldown assay (Fig. 4*A*). βarr1 weakly interacts with both SH3 and SH1 domains of Src, which is consistent with previously published data on βarr1–Src interactions in cells and *in vitro* (12, 13). Interestingly, the SH3 domain of Src binds tighter to active βarr1 (βarr1–V2Rpp), whereas the SH1 domain interacts more strongly with the inactive form of βarr1 (Fig. 4*A*). To understand which of these interactions contribute to the allosteric activation of Src, we performed a competitive colorimetric kinase assay. In this assay we tested the ability of βarr1–V2Rpp to activate Src in the presence of an excess of either purified SH3 domain or a kinase dead mutant of the SH1 domain D386N (SH1 KD). The presence of SH1 KD does not impact the ability of βarr1–V2Rpp to activate Src (Fig. 4*B*). In contrast, an excess of SH3 domain completely blocks the βarr1-mediated activation of Src (Fig. 4*B*) suggesting that the SH3 domain binds to βarr1 and thus interferes with the mechanism of activation through direct competition with Src. Thus, the activation of Src by β-arrestins requires its interaction with the SH3 domain of the enzyme.

## Discussion

Arrestins play a plethora of roles in GPCR signaling. In addition to receptor desensitization, internalization, and intracellular trafficking, arrestins also function as independent signal transducers (reviewed in Ref. 4). However, the precise mechanisms of signal transduction via arrestins remain elusive. Here, we demonstrate that GPCR-activated βarr1 exerts direct allosteric activation of the proto-oncogene kinase Src *in vitro*.

βarr-mediated effects on Src and extracellular signal regulated kinases (ERK1/2) have been explored in two recent studies (15, 16). In particular, it was shown that Src activation downstream of dopamine D1 receptor in HEK 293 cells solely depends on βarr2, whereas ERK1/2 activation involves both G-protein and βarr2 (16). Yang *et al.* (15) showed that βarr1 and GRK6-phosphorylated β2-adrenergic receptor–βarr1 complexes promoted Src activity *in vitro*. These studies, however, do not address the structural and conformational basis for the βarr-mediated allosteric activation of these enzymes. Here, we tested the contribution of five different conformational arrangements of βarr1 and GPCR–βarr1 complexes to the activation of Src (Figs. 3*A* and 4*C*). With respect to receptor engagement these are: 1) fully engaged (tail-and-core of receptor are bound and βarr1 is membrane-anchored (M2V2–βarr1); 2) fully receptor engaged βarr1-deficient in membrane interaction (M2V2–βarr1DDD); 3) partially receptor engaged (tail-bound) membrane anchored (M2V2–βarr1ΔFL); 4) only receptor tail-bound (βarr1–V2Rpp); and 5) inactive βarr1: βarr1, βarr1–V2Rnp, and M2V2np–βarr1. Apart from inactive βarr1, all conformations, including βarr1–V2Rpp, activate Src to a similar level, suggesting that the βarr1-mediated allosteric activation of Src depends only on the receptor tail-bound conformation of βarr1 and does not require its interaction with either the receptor core, or the membrane. Importantly, these findings suggest that initiation of signaling via βarrs may precede the termination of G-protein–mediated signaling, which requires βarr interaction with the receptor core. These results are also consistent with previously published data showing that the tail conformation of the β2V2–βarr1 complex retains the ability to mediate receptor internalization, a process known to be Src-dependent (11, 13, 24). Moreover, the interactions of βarr1 with the core of β2V2 and V2 receptors are also dispensable for ERK2 binding and activation (24, 25). Interestingly, in contrast to the previously published work (15), we did not observe a statistically significant activation of Src in the presence of inactive βarr1. This result can be explained by a different βarr1:Src ratio (5:1 in our study *versus* 6:1 in Ref. 15) and slightly different conditions of the experiment. In addition, in the previous study (15) Src activity was determined by fitting the initial rate to the Michaelis-Menten equation to obtain *K_m_* and *k*_cat_, whereas we used the initial velocity to monitor the lag phase, similarly to previously published papers on activation of Src-family kinases (21, 22).

Intriguingly, we observed identical allosteric effects on Src by βarr1 stimulated by two different receptors, M2V2 and β2V2, sharing the same phosphorylated V2 receptor tail and thus mimicking class B GPCRs. As shown previously (12, 15), WT β2-adrenergic receptor, a class A GPCR, can also activate Src through βarr1 in an agonist and phosphorylation-dependent manner. These findings further buttress the conclusion that it is the phosphorylated receptor C terminus that orchestrates βarr1-mediated Src activation. Moreover, the phosphorylation pattern in the receptor tail is known to affect βarr-mediated recruitment of Src (15). In our study, nonphosphorylated M2V2np did not activate Src through βarr1 (Fig. 3*E*). These results support the barcode hypothesis, the notion that the receptor phosphorylation pattern induces a range of specific conformations of βarr1 that direct βarr1-mediated signaling (26, 27). It is therefore tempting to speculate that a receptor tail with a different phosphorylation pattern might elicit a different outcome on Src recruitment and activation even for the same receptor.

We found that GPCR-activated βarr1 reduces the lag phase in Src activation by promoting its trans-phosphorylation (Fig. 2, *E* and *F*). Furthermore, we showed that allosteric activation of Src requires the interaction of βarr1 with the SH3 domain of the enzyme (Fig. 4*B*). The most plausible mechanism of the activation is therefore a disruption by βarr1–SH3 interactions of Src intramolecular contacts that normally constrain the activity of the kinase (Fig. 4*C*). SH3 domains recognize left-handed type II polyproline sequences P*XX*P (where *X* is any amino acid) (28). In an autoinhibited conformation of Src, the SH3 domain interacts with the type II helix of the linker between the SH2 and kinase domains (29) (Fig. 4*C*). This linker does not have the classical P*XX*P signature, therefore, a partner with an optimal polyproline sequence will easily displace the SH2 linker unlocking the autoinhibited conformation (30). This event represents a common mechanism of Src-family kinase activation and has been documented by several structural and biochemical studies (31, 32).

βarr1 has three P*XX*P sequences (^88^PPAP^91^, ^121^PNLP^124^_,_ and ^175^PERP^178^), and previous studies have demonstrated that mutations of Pro-91 and Pro-121 drastically reduced βarr1-Src interactions and kinase activation (12, 15). It is currently unknown whether one particular site is involved in the interaction or they are inter-changeable. In a computationally generated docking model of GPCR–β-arrestin–Src complex SH3 domain is positioned in the pocket between both Pro-91 and Pro-121 polyproline motifs of βarr1 (33). Intriguingly, none of the proline sites in βarr1 represent a canonical sequence for the SH3 domain of Src that requires consensus sequences R*XX*P*XX*P or P*XX*P*X*R, in which positively charged arginine is important for high affinity binding (28, 34, 35). Perhaps, due to the dynamic nature of the GPCR–βarr1–Src signaling module low affinity interactions between SH3 and βarr1 are preferred. Further structural studies will shed light on the detailed molecular mechanism of Src recruitment and allosteric activation by βarrs.

In conclusion, we demonstrate that binding of βarr1 to the phosphorylated receptor tail instigates a distinct βarr1-mediated signaling pathway via allosteric activation of Src. A combination of biochemical approaches used in this study can easily be applied to explore allosteric activation of other downstream targets by βarr *in vitro*. Taken together, our findings represent an important step forward toward understanding more broadly the mechanisms of signal transduction via βarrs.

## Experimental procedures

### Molecular biology

Constructs expressing WT (residues 2-418) and a minimal cysteine (C59A, C125S, C140I, C150V, C242V, C251V, and C269S) and truncated (βarr1–MC-393) variants of rat βarr1 (17), Fab30 (36), human FLAG-M2 muscarinic and FLAG-β2-adrenergic receptors with C-terminal sortase ligation consensus sequence (LPETGGH) and His_6_ tag (17) have been reported previously. pHH0103_SRC-1/1-IS2/2 plasmid used for the expression of the human Src SH3 domain (residues 87-144) was a gift from Sachdev Sidhu (Addgene plasmid 91251, RRID:Addgene_91251) (37). Plasmids expressing WT chicken c-Src construct that contains the SH3, SH2, and SH1 kinase domains (residues 83-533, Src), kinase domain of WT chicken c-Src (residues 251-533, SH1), and YopH phosphatase were generous gifts from John Kuriyan. Mutations were introduced using QuikChange II site-directed mutagenesis kit (Agilent) and verified by Sanger sequencing.

### Protein expression and purification

The expression and purification of Src(83-533), SH1(251-533), and SH3(87-144) domains from *Escherichia coli* are described in details elsewhere (37, 38). Briefly, Src(83-533) and SH1(251-533) were co-expressed with YopH phosphatase and purified by immobilized metal ion affinity chromatography, anion exchange chromatography, and size-exclusion chromatography (SEC). SH3 was purified by immobilized metal ion affinity chromatography and SEC. WT βarr1 and its variants and Fab30 were expressed and purified as described previously (39, 40). The expression and purification of M2 receptor and β2-adrenergic receptors containing an N-terminal FLAG tag, C-terminal sortase ligation consensus sequence (LPETGGH), and His_6_ tag are described in details elsewhere (9, 17).

### Sortase ligation reactions and HDL reconstitution

Sortase ligation reaction and receptor reconstitution in high density lipoproteins (lipid nanodiscs) are described previously (9, 17). In brief, detergent-solubilized receptor (10 μm) was incubated with a synthetic GGG-V2Rpp (GGG-ARGRpTPPpSLGPQDEpSCpTpTApSpSpSLAKDTSS) (50 μm) or nonphosphorylated GGG-V2Rnp (50 μm) and 2 μm sortaseA in buffer containing 20 mm HEPES, pH 7.4, 100 mm NaCl, 0.1% *n*-dodecyl-β-d-maltoside, 0.01% cholesteryl hemisuccinate, and 5 mm CaCl_2_ overnight at 4 °C. Unligated receptor (containing C-terminal His-tag) and sortase were removed with Talon resin (Invitrogen). Prior to reconstitution, M2V2 and β2V2 receptors (5 μm) were incubated at 4 °C for 30 min with 2-fold molar excess of atropine or ICI-118551, respectively, and then for 1 h with 80 μm membrane scaffold protein (MSP) MSP1D1E3 and a 3:2 molar ratio of 8 mm 1-palmitoyl-2-oleoyl-glycero-3-phosphocholine with 1-palmitoyl-2-oleoyl-*sn*-glycero-3-phospho-(1′-rac-glycerol). Subsequently Bio-Beads (Bio-Rad) were added (0.5 mg/μl of reconstitution volume) and incubation continued overnight at 4 °C with rotation. After reconstitution, HDL receptors were purified by M1-FLAG and SEC.

### Radioligand-binding assays

Saturation and competition radioligand-binding assays were performed at HDL-reconstituted M2V2 and β2V2 (17, 18). All binding assays were carried out until equilibrium at room temperature in a buffer composed of 20 mm HEPES, pH 7.4, 100 mm NaCl, 0.2 mg/ml of BSA and 0.18 mg/ml of ascorbic acid. For saturation ligand bindings a serial dilution of [^3^H]NMS (82 Ci/mmol; PerkinElmer) for M2V2 or ^125^I-CYP (2,200 Ci/mmol; PerkinElmer) for β2V2 was used. Nonspecific radioligand binding was assessed in parallel by including saturating concentrations of cold competitors, atropine (10 μm) for M2V2 and propranolol (20 μm) for β2V2. For competition bindings, the displacement of [^3^H]NMS (1 nm) and ^125^I-CYP (60 pm), at respective receptors, was determined by a serially diluted dose of cold agonist (iperoxo at 10**^−^**^5^ to 10**^−^**^12^m; isoproterenol at 10^−4^ to 10**^−^**^11^m) or antagonist (atropine at 10^−6^ to 10^−13^m; ICI-118551 at 10^−5^ to 10**^−^**^12^m), in the absence or presence of βarr1 at 1 μm (6, 17). Binding reactions were harvested onto glass-fiber filters (GF/B), pre-soaked with 0.3% (v/v) polyethyleneimine in deionized water using a 96-well Brandel harvester. Bound [**^3^**H] was extracted overnight with 5 ml of scintillation fluid and quantified using a Liquid Scintillation Analyzer Tri-Carb 2800TR (PerkinElmer), whereas bound ^125^I was measured using a 2470 Wizard2^TM^ 2-Detector Gamma Counter (PerkinElmer). Binding data were analyzed and plotted in GraphPad Prism using a nonlinear regression curve fit and a one-site specific binding equation to derive the estimates of the apparent maximum specific binding (*B*_max_), equilibrium binding constant (*K_d_*) and IC_50_ for respective conditions. Statistical comparisons of respective IC_50_ values were done by one-way analysis of variance (ANOVA) followed by a Bonferroni's multiple comparison post test and significance was determined at *p* < 0.05.

### Preparation of βarr1–V2Rpp, M2V2–βarr1, and β2V2–βarr1 complexes

To prepare βarr1–V2Rpp complex, βarr1 was incubated with 2-fold molar excess of V2Rpp and Fab30 for 1 h at room temperature and then the complex was purified by SEC in 20 mm HEPES, pH 7.5, 150 mm NaCl, and 1 mm tris(2-carboxyethyl)phosphine hydrochloride (TCEP). To prepare M2V2–βarr1 and β2V2–βarr1 complexes, M2V2 (20 μm) and β2V2 (20 μm) receptors were preincubated with 5-fold molar excess of iperoxo or BI-167107, respectively, for 20 min on ice. After incubation, 20 μm βarr1 and 20 μm Fab30 were added and the mixture was incubated for 1 h on ice.

### Continuous colorimetric kinase assay

Continuous colorimetric kinase assay was performed as previously described (19). All reactions (200 μl) contained Src, 100 mm HEPES, pH 7.5, 150 mm NaCl, 5 mm MgCl_2,_ 1 mm phosphoenolpyruvate, 0.3 mm NADH, 0.25 mm optimal Src peptide (AEEEIYGEFEAKKKK), 2 mm sodium orthovanadate, 1 mm TCEP, 0.005% Triton X-100, 4 units of pyruvate kinase, and 6 units of lactic dehydrogenase. The concentration of Src in all experiments was 25 nM and the concentration of βarr1 or GPCR–βarr1 complex was 125 nm unless stated otherwise. In reactions containing βarr1, βarr1–V2Rpp, or GPCR–βarr1 complexes, the reaction mixture was incubated for 1 h on ice. Reactions were started by the addition of ATP to a final concentration of 0.1 mm, and the decrease in NADH absorbance was monitored over 40 min at 25 °C using a CLARIOstar microplate reader (BMG Labtech). The initial velocity of the reaction (V_0_) was determined using a nonlinear regression curve fit in GraphPad Prism software. The change in absorbance was then converted to the product concentration using the Beer-Lambert law and to the amount of product formed in the reaction volume per minute. Statistical comparisons were determined by one-way ANOVA followed by a Dunnett's multiple comparison test.

### Src autophosphorylation assay

Src autophosphorylation reactions were performed as previously described (21). Src was diluted to 12.5 nm in a buffer containing 100 mm HEPES, pH 7.5, 150 mm NaCl, 5 mm MgCl_2_, 20 μg/ml of BSA, 2 mm sodium orthovanadate, and 1 mm TCEP. In reactions containing 125 nm M2V2–βarr1 complex, the reaction mixture was incubated for 1 h on ice before adding ATP. The reactions were initiated by addition of ATP to a final concentration of 0.1 mm and carried out on ice. At various time points, 50-μl aliquotes of the reaction were quenched with 15 μl of 4× SDS loading buffer and subjected to SDS-PAGE and Western blotting. The active form of Src was detected with anti-Src (phospho-Y418) antibody (Abcam, ab4816, 1:5000 dilution). The total Src was detected by anti-Src antibody (EMD Millipore 05-184, 1:2000 dilution) on a separate SDS-PAGE gel. The optical density of the bands was quantified in ImageJ and statistical differences were determined by Mann-Whitney test in GraphPad Prism software.

### M1-FLAG pulldown assay

To test the binding of βarr1 to M2V2 and M2V2np, 10 μm of the receptor was preincubated with a 5-fold molar excess of iperoxo and the positive allosteric modulator LY211,960 for 30 min on ice and then with a 2-fold molar excess of βarr1 and Fab30 for 2 h on ice. 50 μl of M1-FLAG resin equilibrated in 20 mm HEPES, pH 7.5, 100 mm NaCl buffer was added thereafter, and the mixture was rotated for 1 h at 4 °C and then additional 30 min after adding 2 mm CaCl_2_. After incubation the M1-FLAG beads were collected by centrifugation and washed with 20 mm HEPES, pH 7.5, 100 mm NaCl, 2 mm CaCl_2_ buffer three times. The proteins were eluted with 0.2 mg/ml of FLAG-peptide and 5 mm EDTA in 20 mm HEPES, pH 7.5, 100 mm NaCl buffer. Receptor glycosylation was removed by incubation with a 1:10 protein ratio of peptide:*N*-glycosidase F to the receptor for 60 min at room temperature in the presence of 1% Nonidet P-40, 0.5% SDS, 40 mm DTT. The samples were subjected to SDS-PAGE, visualized by Instant Blue Coomassie stain (Expedeon), and quantified by ImageJ.

### GST-pulldown assay

For detection of βarr1 binding to GST-SH3, 20 μm βarr1 was preincubated with 3-fold molar excess of V2Rpp and Fab30 for 1 h at room temperature, then 10 μm GST-SH3 was added and incubation continued for another 1 h. For detection of SH1 binding to GST-βarr1, 10 μm GST-βarr1 was preincubated with 3-fold molar excess of V2Rpp for 1 h at room temperature, then 20 μm SH1 was added and incubation continued for another 1 h. 50 μl of GST beads (GoldBio) equilibrated in 20 mm HEPES, pH 8.0, 150 mm NaCl buffer were added thereafter, and the mixture incubated for 1 h at room temperature with rotation. After incubation the GST beads were collected by centrifugation and washed with 20 mm HEPES, pH 8.0, 150 mm NaCl buffer three times. The proteins were eluted from GST beads with 40 μl of 20 mm reduced GSH in 20 mm HEPES, pH 8.0, 150 mm NaCl buffer, then mixed with 4× SDS loading buffer, subjected to SDS-PAGE, and Western blotting and detected by EMD Millipore 05-184 antibody (SH3, 1:5000 dilution), Abcam ab1187 anti-His_6_ tag antibody (SH1, 1:5000 dilution), and Cell Signaling Technology 30036S antibody (βarr1, 1:2000 dilution).

## Data availability

All data presented are available upon request from Robert J. Lefkowitz (lefko001@receptor-biol.duke.edu).
