# Supplementary material for: Allosteric activation of proto-oncogene kinase Src by GPCR–beta-arrestin complexes
Source: J Biol Chem. 2021 Jan 13;295(49):16773–84. doi: 10.1074/jbc.RA120.015400 (PMC7864071; doi:10.1074/jbc.RA120.015400)
Supplement: Supplementary file 1 [file mmc1.pdf]

Supporting information for

Allosteric activation of proto-oncogene kinase Src by GPCR–beta-arrestin complexes

Natalia Pakharukova, Ali Masoudi, Biswaranjan Pani, Dean P. Staus, Robert J. Lefkowitz

Table S1
Figure S1
Figure S2

Table S1. Initial velocity of peptide phosphorylation (V0) by Src in the presence of βarr1 and GPCR–βarr1 complexes measured by continuous kinase colorimetric assay. Mean ±SD of five independent experiments are shown (Src: 9 independent experiments). ND – nanodisc

**Experiment**	**V0, nmole min^{-1}**
Src	0.56 ±0.16
SH1	3.16 ±0.45
Src+ND	0.58 ±0.23
Src+βarr1+ND	0.44 ±0.24
Src+M2V2	0.33 ±0.08
Src+M2V2–βarr1	1.1 ±0.31
Src+M2V2–βarr1ΔFL	0.82 ±0.45
Src+M2V2–βarr1DDD	1.1 ±0.25
Src+β2V2	0.34 ±0.11
Src+β2V2–βarr1	0.88 ±0.37
Src+β2V2–βarr1 (no agonist)	0.89 ±0.43
Src+β2V2–βarr1ΔFL	0.79 ±0.11
Src+β2V2–βarr1DDD	0.88 ±0.14
Src+βarr1–V2Rpp	1.1 ±0.24
Src+βarr1–V2Rnp	0.68 ±0.28
Src+M2V2np–βarr1	0.46 ±0.16

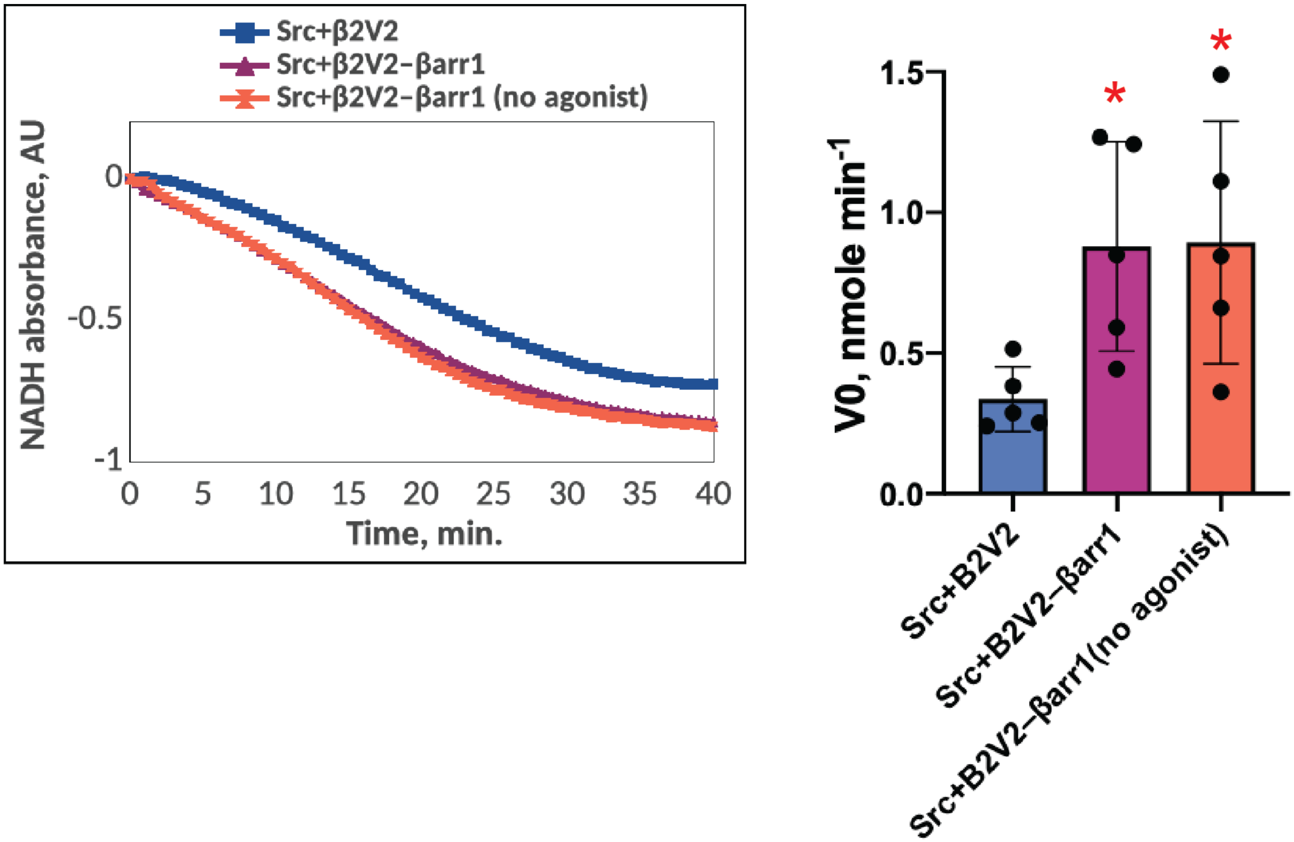

Figure S1: C-terminal tail phosphorylated β2V2 instigates βarr1-mediated activation of Src in the absence of agonist. Left panel: representative progress curves of NADH oxidation coupled to peptide phosphorylation by Src as measured by continuous kinase colorimetric assay. **Right panel:** initial velocity of peptide phosphorylation (V0). Individual data, mean ±SD of five independent experiments are shown. Statistical differences were determined by one-way ANOVA and Dunnett's multiple comparison test (* - $P < 0.05$).

Optimal Src peptide (AEEEIYGEFEAKKKK) is used at a concentration of 250 µM and Src was used at a concentration of 25 nM. β2V2–βarr1 complexes were used at a concentration of 125 nM and are additionally stabilized by a synthetic antibody fragment Fab30 (125 nM). To reproduce the exact conditions of the experiment with GPCR–βarr1 complexes, β2V2 and Fab30 were added to Src at a concentration of 125 nM. β2V2–βarr1: β2V2 was activated by BI-167107; β2V2–βarr1(no agonist): no agonist stimulation of the receptor.

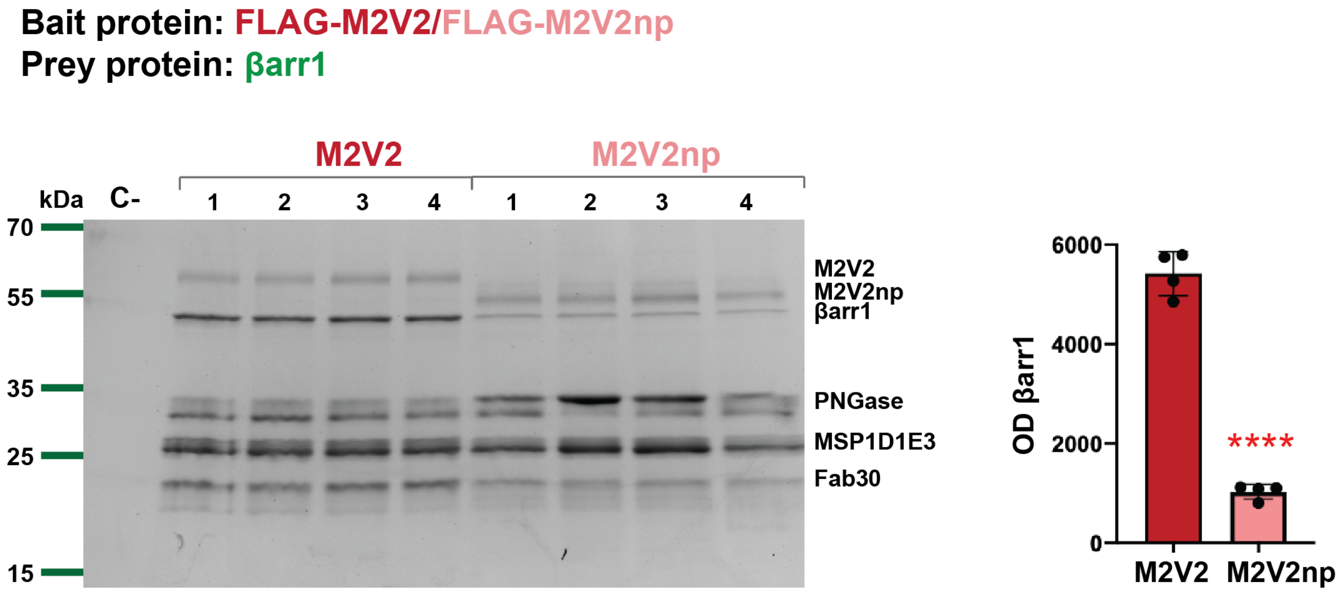

Figure S2: M2V2 with non-phosphorylated tail (M2V2np) binds significantly smaller amount of βarr1 comparing to tail-phosphorylated M2V2. Left panel: Coomasie blue-stained gel of M1-FLAG-pull-down assay of FLAG-M2V2, FLAG-M2V2np and βarr1. All four independent experiments are shown. C- represents a M1-FLAG-pull-down of βarr1 in the absence of receptor. **Right panel:** optical density of βarr1 in M1-FLAG-pull-down assay. Individual data, mean ±SD of four independent experiments are shown. Statistical differences were determined by t-test (* - $P < 0.0001$).
